# Post-Operative Treatment of Six Cases of Abscess of the Appendix, with Some Observations on the Use of Large Doses of Strychnin for Ileus

**Published:** 1903-06

**Authors:** R. M. Harbin

**Affiliations:** Rome, Ga.


### POST-OPERATIVE TREATMENT OF SIX CASES OF ABSCESS OF THE APPENDIX, WITH SOME OBSERVATIONS ON THE USE OF LARGE DOSES OF STRYCHNIN FOR ILEUS.*

By R. M. HARBIN, M.D.,

ROME, GA.

The operation of opening and draining an abscess of the appendix is comparatively simple, and there is no question as to the technique of the procedure. The successful issue of the case, however, depends largely on the after-treatment.

The more radical operation is advised by a few and consists of dissecting out the entire infected area, but more is gained by the conservative method, in which the efforts of nature are utilized in walling off infections of the peritoneum. Few advantages are offered by the radical operation, while the dangers are much increased, and it is better to do a secondary operation, which will rarely be needed.

After draining these abscesses the restoration of the affected parts is almost complete. A writer[1], in referring to secondary operations, remarks: "Indeed, it is a matter for surprise to find so little

*Read at meeting of Medical Association of Georgia, April 15, 1903, Columbus, Ga.

change in the normal condition of the parts in many cases. The safety of treating without removing the appendix is shown by the low mortality. There were two deaths in seventy-four cases."

Berry[2] states that out of forty-one cases treated by the conservative method, only two died; while in thirty-nine radical operations thirty-two died. The danger of recurrence is very slight after incision and drainage, and is estimated at 5.5 per cent. by Wallace.[3]

After the abscess is opened and pus mopped away, a cautious search for foreign bodies should be made, but few will be found.

Iodoform gauze is preferable for packing except in children, who occasionally show marked idiosyncrasy to iodoform poisoning. A strip one to four inches wide should be carried to the bottom of the abscess cavity with the finger, having previously packed some gauze around the wound to protect the peritoneum. The less manipulation the better, as great harm can result. Irrigations and injections are inadvisable, as fluids for this purpose will spread infection by breaking down nature's protective adhesions. After mopping away all the pus, the capillarity of the gauze secures ample drainage. The abscess cavity should be packed to about three fourths its fullness as nearly as can be determined, and the exit still more loosely packed. The gauze should also be packed around the peritoneal opening to keep away knuckles of intestines.

At the end of forty-eight hours the gauze has adhered to the peritoneum, thus making a closed channel for the escape of pus. Over this dressing sterile gauze and cotton may be applied, held by an adhesive strip around the abdomen and over this an abdominal cloth bandage. Whether the abscess wall be adherent to the abdominal parietes or not, the technique is not materially altered.

It is by far preferable to use morphine if the patient's comfort demands it, especially in children. The drug lessens vomiting and circumscribes the area of infection by lessening peristalsis. If there should be shock, strychnin is added. Nothing should be given by the mouth for twelve hours, and then only a little hot or cold water. If there should be decided shock an enema of a pint of normal saline solution, to which is added strong coffee, is ordered. Six hours later a nutrient enema is advisable, consisting of one half ounce liquid peptonoids and one whole egg beaten together, and this repeated as needed.

The successful issue of the case depends largely on moving the bowels, and at the end of forty-eight hours measures for that purpose should be applied. Adhesions having walled off the infection, peristalsis should be aroused to free the intestines from newly-formed adhesions and prevent symptoms of ileus.

As a preliminary, strychnin should be given in large doses four hours previous to the purgative remedies, and the dose should be gr. $\frac{1}{15}$ every two to four hours, hypodermically, until the bowels are moved. The dosage for children should be proportioned to the age. The effect in two cases reported was very marked, and without it other remedies would probably have availed nothing.

The physiologic effects of strychnin commend it as being a rational remedy in these cases, and should be administered hypodermically in maximum doses. Peristalsis of the intestines is hindered by adhesions and the nerve supply paralyzed by toxins.

Shoemaker says of the use of strychnin: "Peristalsis is increased and the bowels somewhat loosened."

As a purgative, calomel serves the best purpose, being less liable to be vomited, and should be given in half to one grain doses with bicarbonate of sodium every hour, until three to five grains are taken. The powders are ordered with a teaspoonful of water, and if vomited, repeat in a half hour. If this routine fails castor oil may be tried.

Recourse is usually had to the various enemata, which may be used seriatim. My preference is for the following: Half gallon of soapsuds water with half ounce of turpentine, which should be mixed by rinsing a cloth saturated with the turpentine over a large bar of soap. Also may be tried half gallon of warm water to which one to three drachms of ox-gall is added. An infusion of flaxseed, to which turpentine and olive oil is added, may be tried, and warm water with glycerine and epsom salts often serves a good purpose. Drastic measures are only indicated in diffuse or general peritonitis.

After the bowels have been moved the dressing should be changed and wound repacked, and may be repeated according to the amount of discharge.

The diet should consist of tea, coffee, meat broth, corn-meal gruel, etc. No milk should be given undiluted, as there is danger of the formation of scybala.

Large abscesses require three to eight weeks for recovery. The after-treatment of cases of rupture and general peritonitis is not materially different except that more drastic purgation is needed. In these cases the first danger is toxemia from peritoneal inflammation, and the next are the symptoms of primary ileus. Fecal fistula is not an unfavorable complication and will require irrigation. Hernia frequently develops after these suppurative cases have healed, but spontaneous recovery may take place, especially in children.

CASE 1.—W. H., aged twenty six, a machinist, had previously had good health and gave no history of any trouble in the region of the appendix. One week before I saw him he had an acute indigestion, which was relieved by a purge. A slight fever, however, persisted and was accompanied by a gradual enlargement in the right iliac fossa. The day before I saw him he had made a railroad journey of seventy-five miles. The enlargement was about the size of a small cocoanut, and there was almost an entire absence of pain. The diagnosis was evident, and the abscess was opened and drained of very fetid pus. The subsequent history was unimportant, and he made a good recovery in six weeks.

CASE 2.—Mrs. Blank, aged thirty three, multipara, was subjected to perineorrhaphy and trachelorrhaphy on October 1, 1901, by Dr. W. P. Harbin. While convalescing she had an attack of intestinal colic from eating sauerkraut. Pain and tenderness on the right side were well marked. She gave a history of having had two attacks of appendicitis, the last being three years previous, in which an operation was advised but was declined. This attack was partially recovered from. In the present attack purgatives were given and followed by satisfactory results, but she continued to vomit and suffer pain in the right side along with an enlargement gradually increasing each day. Temperature varied from normal to 103° F. in the evenings and pulse beats did not exceed 90. The mass now corresponded to the size of a flattened orange.

By the assistance of Drs. Shaw and DeLay an operation was performed on the tenth day of the present attack, and a four-inch incision over the summit of the mass was made parallel to Poupart's ligament. On opening the peritoneum a bulging was observed under the mesentery of the ascending colon and caput coli, both of which seemed abnormally low. An attempt was made to aspirate the mass through the mesentery, but without success. Adhesions were very extensive, binding down about eight inches of the ascending colon. Separation of the adhesions was begun at the bottom of the right iliac fossa in a very slow manner, and extended upwards, hugging the fossa until the mass was reached. This part of the procedure occupied nearly two hours. Much of the enlargement was due to old inflammation exudates, and the abscess proper was not larger than a walnut. After mopping the wound dry a piece of iodoform gauze was carried to the abscess cavity with the finger.

There was considerable shock, but she reacted well in twelve hours. Symptoms of a very diffuse peritonitis developed, the temperature reaching 103° and pulse 130, with vomiting, pain and tympany. At the end of forty-eight hours strychnin was begun hypodermically in doses of 1-15 grain every two hours along with a grain of calomel every hour, until six were taken, two of which were vomited. The various enemata were used, and after twelve hours of strenuous efforts the bowels were moved. The wound was repacked and the discharge became profuse and offensive. In four weeks the wound closed, and she has been well ever since, but she has hernia.

CASE 3.—L. L., a young man seventeen years old, had acute indigestion and colic, March 1, 1902. Ten days later I was asked to see him with Drs. Bradley and Battle, of Cassville, Ga. There was no history of any previous attacks of appendicitis. Purgatives had been given with good results, but pain in the right side, with three or four degrees increase of temperature, persisted along with an indurated mass in the right iliac fossa about the size of a small apple. Laparotomy was done at once, and after separating the adhesions of the caput coli the abscess was ruptured and drained. The abscess was treated in the usual manner, and his recovery was uneventful.

CASE 4.—R. C., a four-year-old boy, ate bananas on May 31, 1902, after which occurred pain in the abdomen and vomiting. Purgatives were inefficient, and three days later I saw him with a temperature of 103° and pulse 120. The facies indicated an extreme amount of depression. Castor oil and calomel purged well but gave little relief. A slight induration in the region of the appendix could be detected and gradually increased until it equalled the size of a lemon six days later. The abdomen was opened and drained of two or three ounces of fetid pus. The wound was packed with iodoform gauze. On the second day a scarlatinal rash with restlessness developed, presumably due to iodoform poisoning, but passed off in twenty-four hours. On the fourth day, while repacking the wound without anesthesia, a false passage was made and resulted in the formation of a secondary abscess. His recovery was uneventful, but had hernia.

CASE 5.—On November 9, 1902, I was asked to see B. G., a healthy girl two years and nine months old, at Shannon, Ga., with Drs. Shaw and Mull. Five days before she had an attack of acute indigestion from eating apples, which caused vomiting, pain and tympany. The various purgatives were given without avail. Tympany was excessive, and no peristalsis could be detected, heart sounds being distinctly audible over the entire abdomen. Temperature changes were inconsiderable and pulse beats were 130 and very tense. A general peritonitis was very evident, and presumably due to a ruptured appendix two or three days before. The abdomen was opened and a sero-purulent discharge was drained away. Adhesions being extensive were separated down to the iliac fossa in the region of the appendix, and the canal was packed with gauze. There was no appreciable shock, and the patient's comfort was greatly enhanced. The usual routine of after-treatment was applied, and on the second day 1-100 grain of strychnin was given hypodermically every two hours, along with ½ grain of calomel every hour. By the use of enemata and a dose of castor oil the bowels moved in twelve hours. In all 1-20 grain of strychnine was taken.

The wound was repacked and this process repeated every second day. On the fifth day a violent colitis developed without assignable cause and was accompanied by a great amount of tenesmus. Strychnin was continued in moderate doses and after two or three days a "strychnin delirium" developed, causing singing, playing, laughing, etc., and was very different from ordinary delirium. When the drug was suspended the delirium subsided. The colitis improved in four or five days but she was very weak. In such a condition it was difficult to nourish her, and in five weeks she died from exhaustion. She may be said to have recovered from general peritonitis primarily.

CASE 6.—A. W., a nine-year-old boy, had had previous attacks of pain in the abdomen. On December 13, 1902, he was indisposed and three grains of calomel were given which quite relieved him. For a week his temperature ranged from normal to 103°, and his illness was diagnosed one of gastro-intestinal intoxication. He was now allowed more food. On December 26, after eating some milk thickened with rice, he had a very acute pain in the abdomen with symptoms of collapse, thready pulse, clammy perspiration, excessive vomiting and a great amount of tympanites. Twenty-four hours later I was asked to see him with Dr. Wicker and found all the above symptoms intensified. Pulse varied from 130 to 150, irregular. A diagnosis of general peritonitis was made, probably due to a ruptured appendix. The abdomen was opened and adhesions found to be general, which were separated down to the region of the appendix. A positive diagnosis could not be made as further manipulations were deemed to be unwise. The wound was packed with sterile gauze which provoked a free serous drainage. The abdominal tension was greatly relieved and in every way his comfort was much increased. At the end of forty-eight hours his bowels moved spontaneously and tympanites was much reduced. The pulse and general condition were much improved. On the eighth day violent colicky pains developed with vomiting. There was evidently intestinal obstruction, and the general condition was so bad operative measures were not advisable and only palliative treatment was applied. The vomit changed from bilious to stercoraceous material. Nutritive enemata kept up his strength. Epigastric distention was so great that gastric lavage was resorted to, giving great relief and was repeated two to three times daily. On the twelfth day after the operation a secondary abscess ruptured through the wound and colon, giving exit to a great amount of fetid pus. He died the next day.

The following deductions may be made :

1. The after-treatment of abscesses of the appendix is the most important part of the procedure.
2. Unnecessary manipulation is harmful and the conservative operation is preferable.
3. Early operation in cases of rupture and with general peritonitis will save some cases, especially children.
4. Strychnin in large doses is a powerful adjuvant to purgative remedies in relieving primary ileus.
5. Iodoform gauze should not be used in children for fear of iodoform poisoning.
6. Anesthetics should be used in repacking the wounds in children.
7. Gastric lavage gives great relief to cases with stercoraceous vomiting.

BIBLIOGRAPHY.

[1] Battle, *Lancet*, January 18, 1902.
[2] Clinical Society of London, March 14, 1902.
[3] St. Thomas Hospital Reports.

